# Supplementary figures and images for: Lipolysis of bone marrow adipocytes is required to fuel bone and the marrow niche during energy deficits
Source: eLife. 2022 Jun 22;11:e78496. doi: 10.7554/eLife.78496 (PMC9273217; doi:10.7554/eLife.78496)

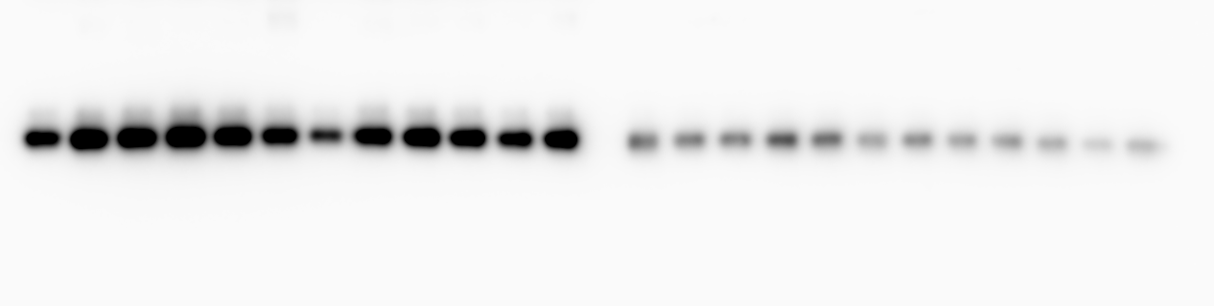

Supplement: Figure 1—figure supplement 2—source data 1. [file elife-78496-fig1-figsupp2-data1.zip › Source data files_Figure 1_figure supplement 2/Figure 1 - figure supplement 2B_2C_Adiponectin.tif]

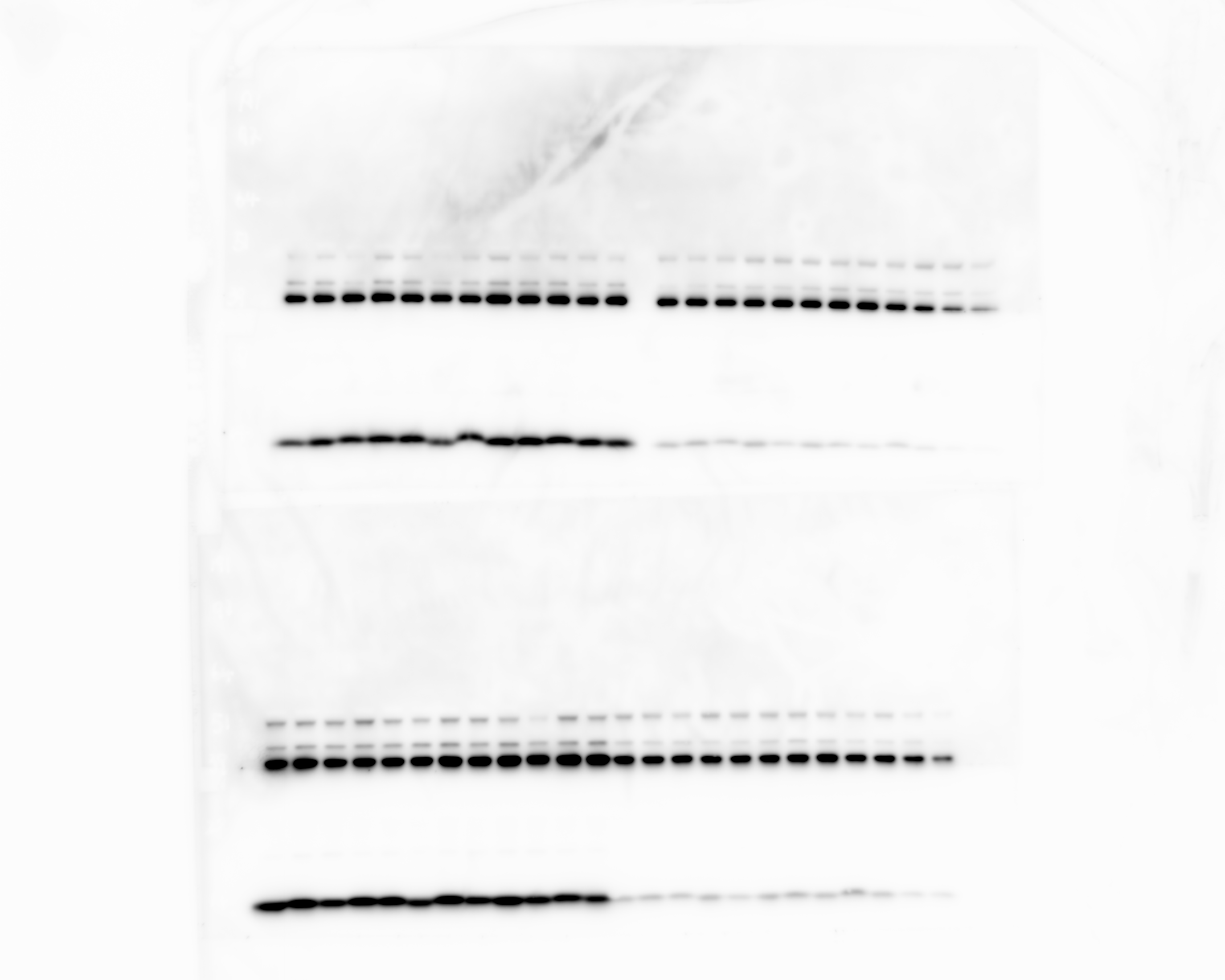

Supplement: Figure 1—figure supplement 2—source data 1. [file elife-78496-fig1-figsupp2-data1.zip › Source data files_Figure 1_figure supplement 2/Figure 1 - figure supplement 2B_2C_FABP4 tubulin and ERK.tif]

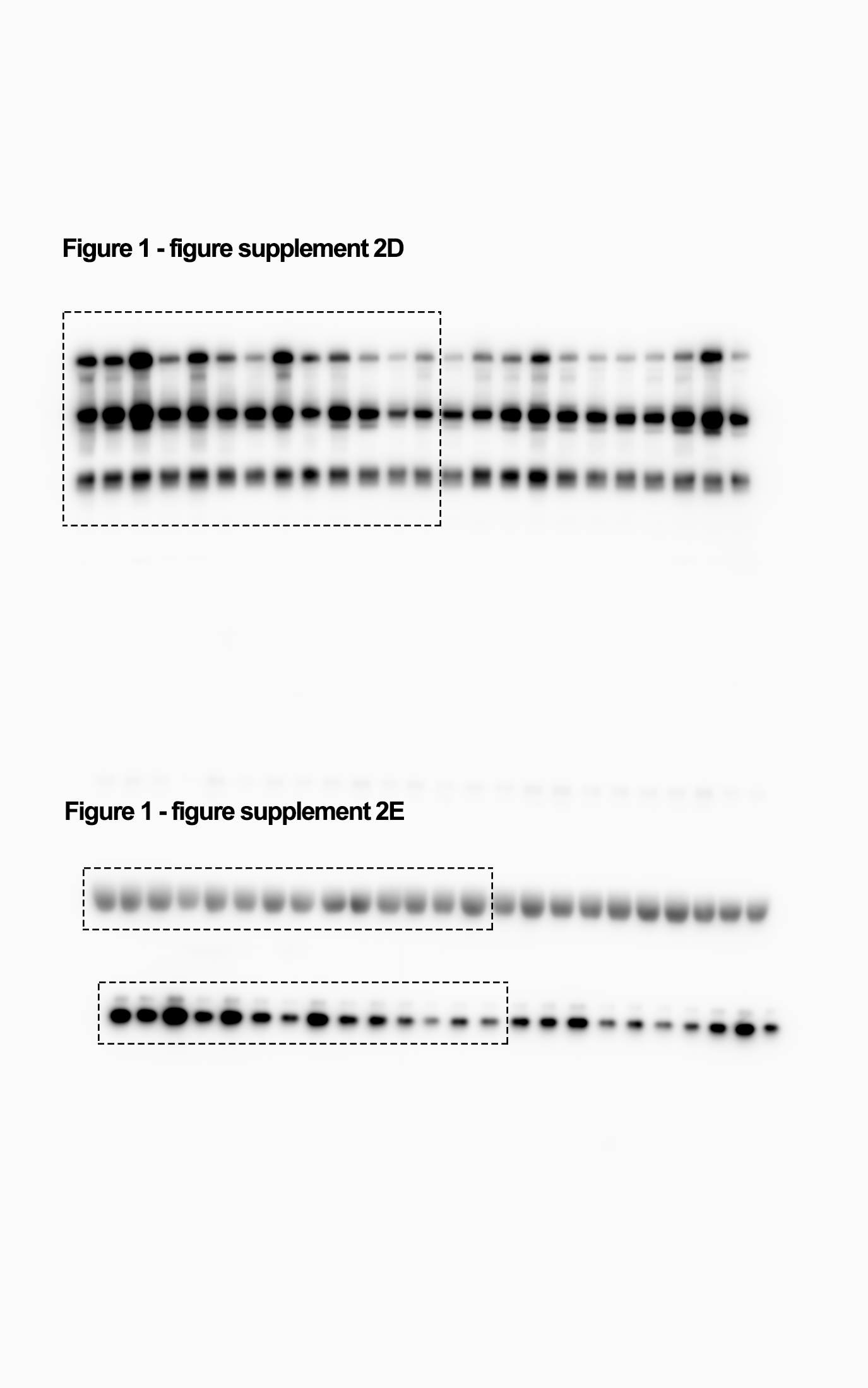

Supplement: Figure 1—figure supplement 2—source data 1. [file elife-78496-fig1-figsupp2-data1.zip › Source data files_Figure 1_figure supplement 2/Figure 1 - figure supplement 2D_Adiponectin and albumin.tif]

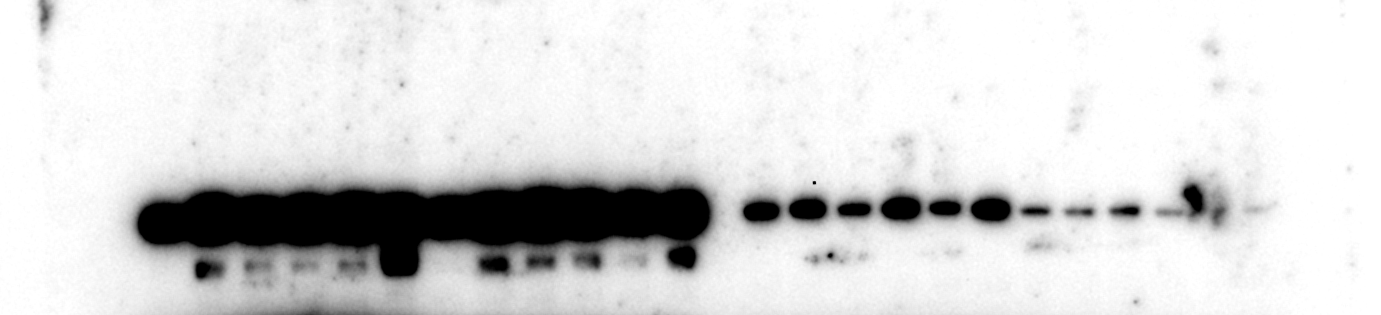

Supplement: Figure 2—source data 1. [file elife-78496-fig2-data1.zip › Source data files_Figure 2 /Figure 2C caudal vertebrae ATGL.tif]

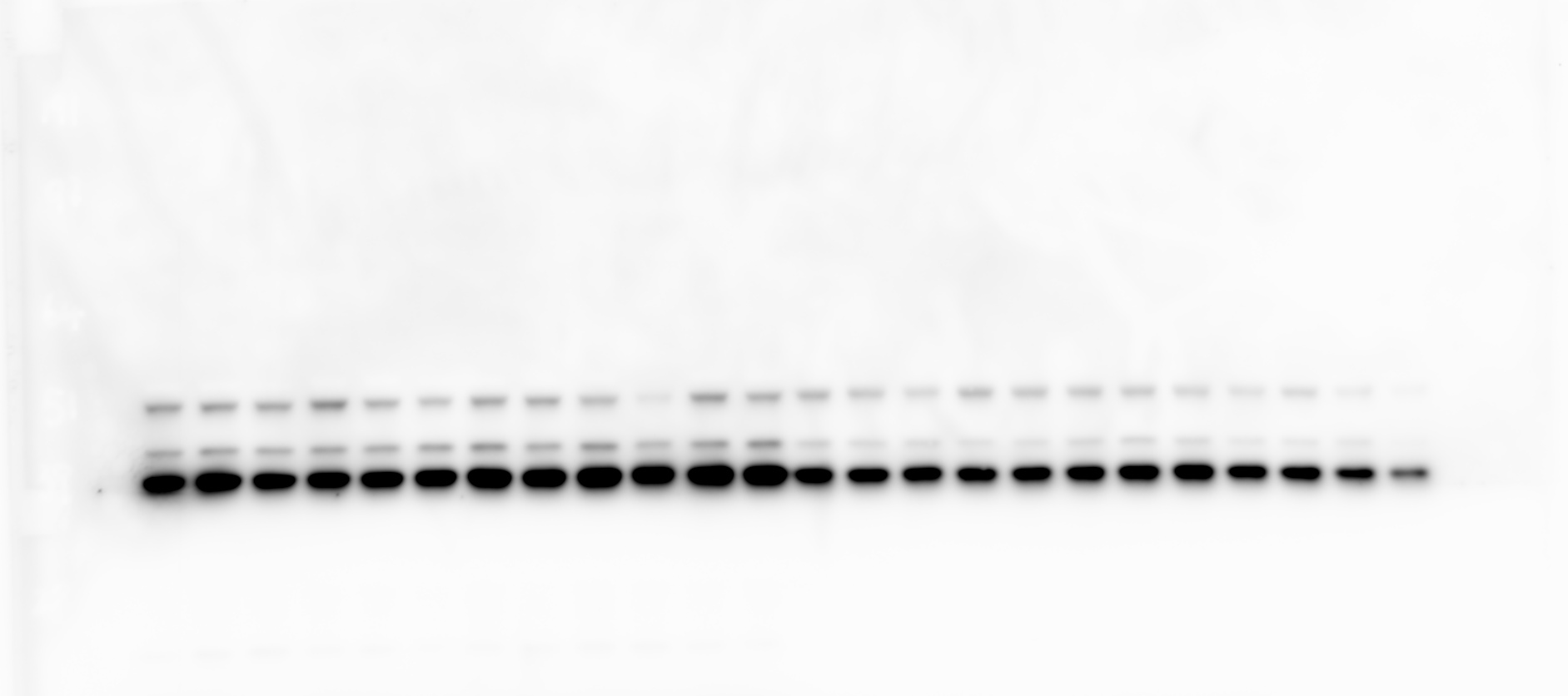

Supplement: Figure 2—source data 1. [file elife-78496-fig2-data1.zip › Source data files_Figure 2 /Figure 2C a-tubulin and ERK.tif]

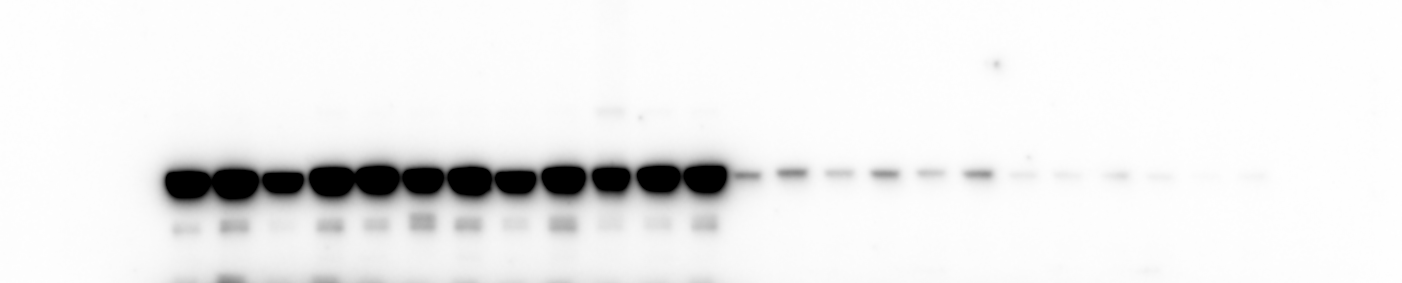

Supplement: Figure 2—source data 1. [file elife-78496-fig2-data1.zip › Source data files_Figure 2 /Figure 2C subcutaneous WAT ATGL.tif]

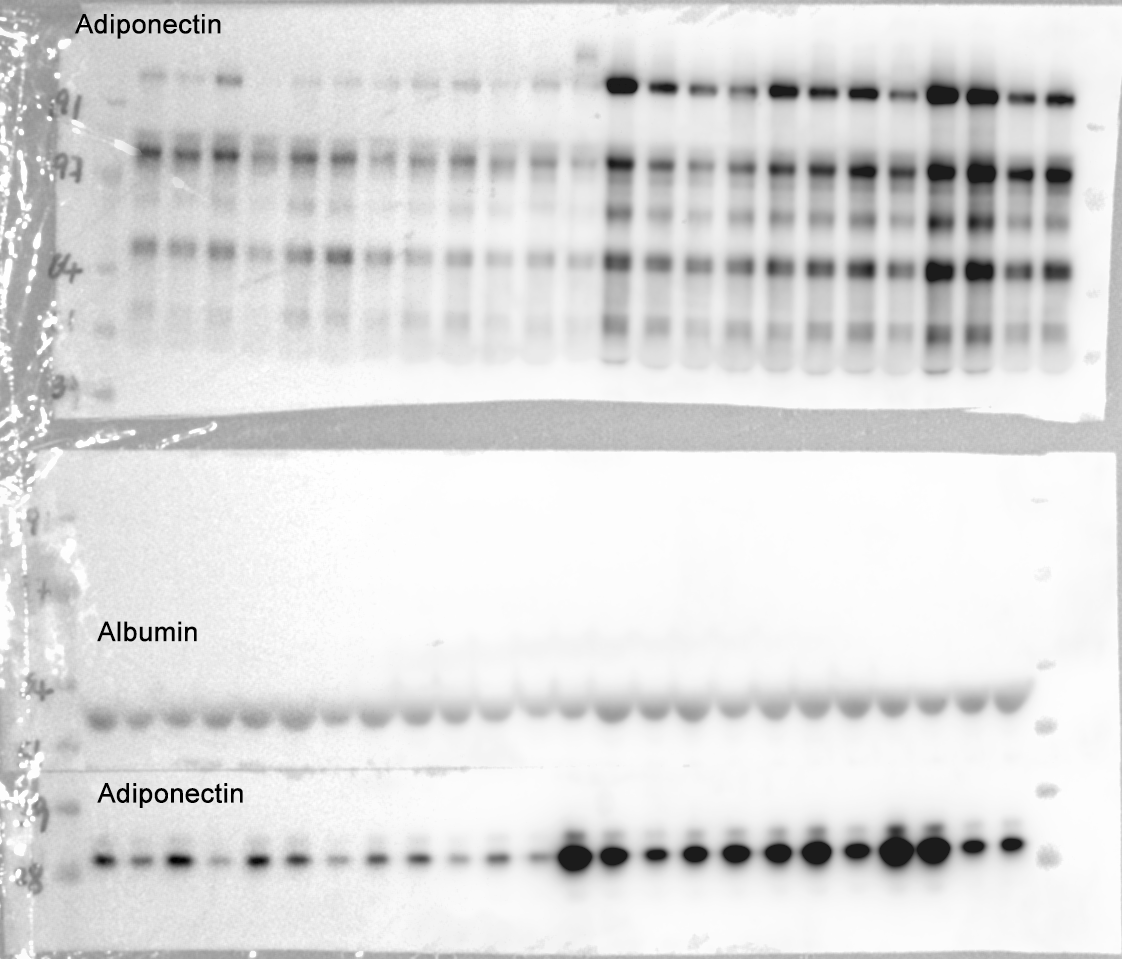

Supplement: Figure 3—figure supplement 1—source data 1. [file elife-78496-fig3-figsupp1-data1.zip › Source data files_Figure 3_figure supplement 1/Source data files_Figure 3_figure supplement 1V.tif]
