# Supplementary material for: Lipolysis of bone marrow adipocytes is required to fuel bone and the marrow niche during energy deficits
Source: eLife. 2022 Jun 22;11:e78496. doi: 10.7554/eLife.78496 (PMC9273217; doi:10.7554/eLife.78496)
Supplement: Supplementary file 1. — Whole blood from male mice at 24 weeks of age fed ad libitum (top) or a 30% CR diet for 6weeks (bottom) was submitted for complete blood counts (CBC). White- and red- blood cell related parameters are shown. Multiple unpaired t tests had been performed crossing all parameters, P values were adjusted for multiple comparisons using Two-stage step-up (Benjamini, Krieger, and Yekutieli) with FDR method. [file elife-78496-supp1.docx]

**Supplementary File 1. BMAd-lipolysis deficiency has subtle effects on circulating mature blood cells.**

| **Ad libitum** | | **BMAd-Pnpla2^+/+^ (n=5)** | | **BMAd-Pnpla2^-/-^ (n=6)** | | |  |
| --- | --- | --- | --- | --- | --- | --- | --- |
| Parameter | Units | Mean | Std | Mean | Std | *P* values | Adjusted *P* |
| WBC | K/uL | 6.39 | 0.90 | 5.04 | 2.53 | 0.265966 | 0.810307 |
| NE# | K/uL | 2.58 | 1.25 | 2.03 | 0.60 | 0.405102 | 0.872916 |
| LY# | K/uL | 3.28 | 1.29 | 2.64 | 1.97 | 0.534720 | 0.888349 |
| MO# | K/uL | 0.43 | 0.21 | 0.30 | 0.12 | 0.264883 | 0.810307 |
| EO# | K/uL | 0.08 | 0.10 | 0.07 | 0.06 | 0.850688 | 0.962075 |
| BA# | K/uL | 0.012 | 0.01 | 0.01 | 0.01 | 0.749046 | 0.962075 |
| NE% | % | 40.52 | 18.02 | 46.88 | 15.88 | 0.555425 | 0.888349 |
| LY% | % | 51.51 | 18.25 | 45.39 | 16.75 | 0.580667 | 0.896460 |
| MO% | % | 6.54 | 2.27 | 6.28 | 1.98 | 0.846152 | 0.962075 |
| EO% | % | 1.25 | 1.42 | 1.31 | 0.74 | 0.934886 | 0.986802 |
| BA% | % | 0.18 | 0.15 | 0.14 | 0.13 | 0.652445 | 0.903165 |
| RBC | M/uL | 9.15 | 1.02 | 8.77 | 0.98 | 0.547578 | 0.888349 |
| HB | g/dL | 14.04 | 1.92 | 13.53 | 1.56 | 0.646503 | 0.903165 |
| HCT | % | 42.68 | 5.60 | 41.32 | 4.55 | 0.674628 | 0.903165 |
| MCV | fL | 46.54 | 1.91 | 47.17 | 1.93 | 0.601371 | 0.901903 |
| MCH | Pg | 15.32 | 0.82 | 15.47 | 0.91 | 0.780413 | 0.962075 |
| MCHC | g/dL | 32.88 | 1.03 | 32.75 | 1.10 | 0.844431 | 0.962075 |
| RDW | % | 19.02 | 0.90 | 18.63 | 0.92 | 0.497192 | 0.888349 |
| PLT | K/uL | 864.8 | 127.15 | 933 | 121.51 | 0.391066 | 0.872916 |
| MPV | fL | 5.18 | 0.63 | 4.7 | 0.51 | 0.209160 | 0.810307 |

| **CR** |  | **BMAd-Pnpla2^+/+^ (n=7)** | | **BMAd-Pnpla2^-/-^ (n=7)** | | |  |
| --- | --- | --- | --- | --- | --- | --- | --- |
| Parameter | Units | Mean | Std | Mean | Std | *P* values | Adjusted *P* |
| WBC | K/uL | 3.98 | 0.93 | 3.56 | 1.02 | 0.436458 | 0.872916 |
| NE# | K/uL | 1.80 | 0.52 | 1.19 | 0.39 | **0.028803*** | 0.576180 |
| LY# | K/uL | 1.89 | 0.78 | 2.13 | 0.67 | 0.548422 | 0.888349 |
| MO# | K/uL | 0.15 | 0.08 | 0.18 | 0.05 | 0.416605 | 0.872916 |
| EO# | K/uL | 0.12 | 0.10 | 0.05 | 0.06 | 0.138249 | 0.810307 |
| BA# | K/uL | 0.03 | 0.03 | 0.01 | 0.02 | 0.167929 | 0.810307 |
| NE% | % | 45.88 | 13.18 | 33.46 | 6.12 | **0.043110*** | 0.576180 |
| LY% | % | 46.94 | 14.20 | 59.62 | 6.87 | 0.054874 | 0.576180 |
| MO% | % | 3.70 | 1.04 | 5.30 | 1.40 | 0.031891 | 0.576180 |
| EO% | % | 2.86 | 2.04 | 1.25 | 1.57 | 0.123874 | 0.810307 |
| BA% | % | 0.62 | 0.64 | 0.36 | 0.54 | 0.427388 | 0.872916 |
| RBC | M/uL | 9.21 | 0.98 | 9.74 | 0.80 | 0.289395 | 0.810307 |
| HB | g/dL | 13.04 | 1.43 | 13.84 | 1.22 | 0.282170 | 0.810307 |
| HCT | % | 42.57 | 4.47 | 45.16 | 3.70 | 0.260511 | 0.810307 |
| MCV | fL | 46.24 | 1.82 | 46.37 | 0.92 | 0.868875 | 0.962075 |
| MCH | Pg | 14.16 | 0.60 | 14.21 | 0.52 | 0.870448 | 0.962075 |
| MCHC | g/dL | 30.63 | 0.66 | 30.66 | 0.79 | 0.939811 | 0.986802 |
| RDW | % | 18.56 | 0.95 | 18.09 | 0.41 | 0.252621 | 0.810307 |
| PLT | K/uL | 947.86 | 124.44 | 838.29 | 185.96 | 0.219485 | 0.810307 |
| MPV | fL | 5.13 | 0.52 | 4.89 | 0.37 | 0.339399 | 0.872916 |

Whole blood from male mice at 24 weeks of age fed *ad libitum* (top) or a 30% CR diet for 6 weeks (bottom) was submitted for complete blood counts (CBC). White- and red- blood cell related parameters are shown. Multiple unpaired t tests had been performed crossing all parameters, *P* values were adjusted for multiple comparisons using Two-stage step-up (Benjamini, Krieger, and Yekutieli) with FDR method.
