# Supplementary material for: Lipolysis of bone marrow adipocytes is required to fuel bone and the marrow niche during energy deficits
Source: eLife. 2022 Jun 22;11:e78496. doi: 10.7554/eLife.78496 (PMC9273217; doi:10.7554/eLife.78496)
Supplement: Supplementary file 2. — Femoral bone marrow cells from male mice at 24 weeks of age fed ad libitum (top) or a 30% CR diet for 6 weeks (bottom) were collected and stained with antibodies for flow cytometry analyses. Mature blood cells and hematopoietic stem/progenitor cells (HSPCs) were counted. Multiple unpaired t tests had been performed crossing all parameters, P values were adjusted for multiple comparisons using Two-stage step-up (Benjamini, Krieger, and Yekutieli) with FDR method. [file elife-78496-supp2.docx]

**Supplementary File 2. BMAd-lipolysis deficiency causes mild changes in bone marrow hematopoietic cells.**

| **Ad libitum** |  | | **BMAd-Pnpla2^+/+^**  **(n=5)** | | | **BMAd-Pnpla2^-/-^**  **(n=6)** | | | |  | |  | | **BMAd-Pnpla2^+/+^**  **(n=5)** | | | **BMAd-Pnpla2^-/-^**  **(n=6)** | | | |  |  |  |
| --- | --- | --- | --- | --- | --- | --- | --- | --- | --- | --- | --- | --- | --- | --- | --- | --- | --- | --- | --- | --- | --- | --- | --- |
| Counts | Units | Mean | | Std | Mean | | Std | *P* values | Adjusted *P* | | Percentage | | Mean | | Std | Mean | | Std | *P* values | Adjusted *P* | | | |
| BMNC | 10^6/ml | 21.89 | | 1.78 | 19.33 | | 3.27 | 0.153 | 0.428 | | \ | | \ | | \ | \ | | \ | \ |  | | | |
| NE# | 10^6/ml | 13.93 | | 1.39 | 11.63 | | 1.73 | **0.040*** | 0.382 | | NE% | | 63.62 | | 3.60 | 60.43 | | 3.66 | 0.181 | 0.428 | | | |
| MO# | 10^5/ml | 0.74 | | 0.23 | 0.69 | | 0.32 | 0.777 | 0.918 | | MO% | | 0.34 | | 0.09 | 0.35 | | 0.16 | 0.904 | 0.949 | | | |
| B cell | 10^5/ml | 5.96 | | 4.46 | 8.55 | | 7.56 | 0.519 | 0.701 | | B cell% | | 2.86 | | 2.40 | 4.11 | | 3.53 | 0.519 | 0.701 | | | |
| T cell | 10^5/ml | 8.46 | | 3.82 | 5.06 | | 2.28 | **0.010*** | 0.428 | | T cell% | | 3.8 | | 1.53 | 2.68 | | 1.35 | 0.229 | 0.433 | | | |
| **HSPCs** |  |  | |  |  | |  |  |  | |  | |  | |  |  | |  |  |  | | | |
| HSC | 10^3/ml | 1.88 | | 1.35 | 1.58 | | 0.96 | 0.677 | 0.921 | | HSC% | | 0.008 | | 0.005 | 0.009 | | 0.006 | 0.774 | 0.921 | | | |
| MPP | 10^3/ml | 2.84 | | 1.80 | 1.96 | | 1.16 | 0.351 | 0.784 | | MPP% | | 0.013 | | 0.008 | 0.010 | | 0.007 | 0.523 | 0.849 | | | |
| GMP | 10^3/ml | 5.36 | | 2.44 | 1.85 | | 1.48 | **0.01*** | 0.242 | | GMP% | | 0.024 | | 0.011 | 0.010 | | 0.008 | **0.037*** | 0.242 | | | |
| PreGM | 10^3/ml | 8.59 | | 6.78 | 7.38 | | 5.06 | 0.742 | 0.921 | | PreGM% | | 0.039 | | 0.031 | 0.039 | | 0.026 | >0.9999 | >0.9999 | | | |
| PreMegE | 10^3/ml | 2.38 | | 2.31 | 2.00 | | 1.34 | 0.740 | 0.921 | | PreMegE% | | 0.011 | | 0.010 | 0.011 | | 0.008 | >0.9999 | >0.9999 | | | |
| PreCFUe | 10^3/ml | 0.35 | | 0.09 | 0.58 | | 0.57 | 0.399 | 0.791 | | PreCFUe% | | 0.002 | | 0.0004 | 0.003 | | 0.003 | 0.482 | 0.849 | | | |
| HPC 1 | 10^3/ml | 5.16 | | 3.68 | 2.83 | | 1.77 | 0.200 | 0.555 | | HPC 1% | | 0.025 | | 0.019 | 0.014 | | 0.007 | 0.217 | 0.555 | | | |
| HPC 2 | 10^3/ml | 1.01 | | 0.64 | 0.39 | | 0.24 | 0.054 | 0.242 | | HPC 2% | | 0.005 | | 0.003 | 0.002 | | 0.001 | 0.045 | 0.242 | | | |

| **CR** |  | **BMAd-Pnpla2^+/+^ (n=6)** | | | **BMAd-Pnpla2^-/-^ (n=7)** | | |  | |  | |  |  | **BMAd-Pnpla2^+/+^**  **(n=6)** | | | | **BMAd-Pnpla2^-/-^ (n=7)** | |  | |
| --- | --- | --- | --- | --- | --- | --- | --- | --- | --- | --- | --- | --- | --- | --- | --- | --- | --- | --- | --- | --- | --- |
| Counts | Units | Mean | Std | Mean | | Std | *P* values | | Adjusted *P* | | Percentage | | | | Mean | Std | Mean | Std | *P* values | | Adjusted *P* |
| BMNC | 10^6/ml | 20.79 | 6.00 | 16.64 | | 2.44 | 0.120 | | 0.227 | |  | | | | \ | \ | \ | \ | \ | |  |
| NE# | 10^6/ml | 9.40 | 3.25 | 5.83 | | 1.44 | **0.023*** | | 0.065 | | NE% | | | | 45 | 4.49 | 34.74 | 5.58 | **0.004*** | | **0.035*** |
| MO# | 10^5/ml | 1.43 | 0.44 | 1.12 | | 0.24 | 0.135 | | 0.227 | | MO% | | | | 6.89 | 0.99 | 6.71 | 0.88 | 0.735 | | 0.686 |
| B cell | 10^5/ml | 41.96 | 21.14 | 52.68 | | 10.73 | 0.262 | | 0.324 | | B cell% | | | | 19.83 | 8.01 | 31.9 | 6.84 | **0.013*** | | 0.057 |
| T cell | 10^5/ml | 10.09 | 6.64 | 7.10 | | 1.578 | 0.270 | | 0.324 | | T cell% | | | | 5.11 | 3.49 | 4.36 | 1.23 | 0.604 | | 0.634 |
| **HSPCs** |  |  |  |  | |  |  | |  | |  | | | |  |  |  |  |  | |  |
| HSC | 10^3/ml | 1.166 | 0.67 | 0.77 | | 0.21 | 0.164 | | 0.733 | | HSC% | | | | 0.057 | 0.032 | 0.05 | 0.015 | 0.614 | | 0.786 |
| MPP | 10^3/ml | 0.93 | 0.31 | 0.79 | | 0.20 | 0.347 | | 0.733 | | MPP% | | | | 0.045 | 0.010 | 0.05 | 0.009 | 0.363 | | 0.733 |
| GMP | 10^3/ml | 6.91 | 3.40 | 4.47 | | 1.11 | 0.099 | | 0.733 | | GMP% | | | | 0.33 | 0.114 | 0.27 | 0.046 | 0.26 | | 0.733 |
| PreGM | 10^3/ml | 16.57 | 4.85 | 14.72 | | 3.48 | 0.441 | | 0.733 | | PreGM% | | | | 0.812 | 0.170 | 0.88 | 0.174 | 0.493 | | 0.733 |
| PreMegE | 10^3/ml | 4.95 | 3.48 | 3.94 | | 1.21 | 0.485 | | 0.733 | | PreMegE% | | | | 0.248 | 0.178 | 0.24 | 0.077 | 0.916 | | >0.9999 |
| PreCFUe | 10^3/ml | 5.28 | 2.81 | 4.28 | | 1.04 | 0.398 | | 0.733 | | PreCFUe% | | | | 0.247 | 0.066 | 0.27 | 0.094 | 0.626 | | 0.786 |
| HPC 1 | 10^3/ml | 6.76 | 4.32 | 5.25 | | 1.66 | 0.409 | | 0.733 | | HPC 1% | | | | 0.33 | 0.202 | 0.33 | 0.136 | >0.9999 | | >0.9999 |
| HPC 2 | 10^3/ml | 16.08 | 12.78 | 10.94 | | 2.90 | 0.320 | | 0.733 | | HPC 2% | | | | 0.8 | 0.652 | 0.68 | 0.255 | 0.661 | | 0.786 |

Femoral bone marrow cells from male mice at 24 weeks of age fed *ad libitum* (top) or a 30% CR diet for 6 weeks (bottom) were collected and stained with antibodies for flow cytometry analyses. Mature blood cells and hematopoietic stem/progenitor cells (HSPCs) were counted. Multiple unpaired t tests had been performed crossing all parameters, *P* values were adjusted for multiple comparisons using Two-stage step-up (Benjamini, Krieger, and Yekutieli) with FDR method.
