# Supplementary material for: Lipolysis of bone marrow adipocytes is required to fuel bone and the marrow niche during energy deficits
Source: eLife. 2022 Jun 22;11:e78496. doi: 10.7554/eLife.78496 (PMC9273217; doi:10.7554/eLife.78496)
Supplement: Supplementary file 3. [file elife-78496-supp3.docx]

**Supplementary File 3. PCR primer list.**

| **Name** | **Sequence** |
| --- | --- |
| *Genotype PCR* |  |
| *Osterix* p1 | CCGCCCCGATCTTCCACT |
| *Osterix* p2 | GTTGCCGGTCCTGTTCACTCTC |
| *Osterix* p3 | TGCTCCCGGCCAGGTTACTA |
| FAC p1 | CACTCTAAATGAACACGTGCTTTCG |
| FAC p2 | ACCGGCATCAACGTTTTCTTTT |
| FAC p3 | CTGGATAGTGAAACAGGGGCA |
| FAC-WT Forward | AGCCCATACACCAGGAGAATCA |
| FAC-WT Reverse | TGTGAAGCCCCCATACCAA |
| FAC-Mut Forward | AGCCCATACACCAGGAGAATCA |
| FAC-Mut Reverse | TTGGCGAGAGGGGAAAGACC |
|  |  |
| *Regular PCR* |  |
| *Pnpla2* Forward | CCAACGCCACTCACATCTACG |
| *Pnpla2* Reverse | ACCCCGGGGCTCCTCTTA |
|  |  |
| *qPCR* |  |
| FAC Original band- Forward | TCGCTAGCTCAATCGCCATCTT |
| FAC Original band-Reverse | GCCACCAGCCAGCTATCAACTC |
| FAC Flipped band- Forward | TGAAGGATGCCCAGAAGGTA |
| FAC Flipped band-Reverse | CGGCAAACGGACAGAAGC |
| *Pnpla2* Forward | CGGCTTCCTCGGGGTCTAC |
| *Pnpla2* Reverse | CGCGCTCATGGCAATCAG |
| *Adipoq* Forward | CATTCCGGGACTCTACTACTTCT |
| *Adipoq* Reverse | GAGGCCTGGTCCACATTCTT |
| *Pparγ* Forward | GCCATTGAGTGCCGAGTCTGT |
| *Pparγ* Reverse | GCATCCGCCCAAACCTGA |
| *Cebpα* Forward | TGGACAAGAACAGCAACGAG |
| *Cebpα* Reverse | TCACTGGTCAACTCCAGCAC |
| *Fabp4* Forward | ATGAAATCACCGCAGACGACA |
| *Fabp4* Reverse | CACGCCTTTCATAACACATTCC |
| *Scd1* Forward | CGTGGGTTGGCTGCTTGTG |
| *Scd1* Reverse | CAGGAGGCCGGGCTTGTAGT |
| *Rpl32a* Forward | GAGCAACAAGAAAACCAAGCA |
| *Rpl32a* Reverse | TGCACACAAGCCATCTACTCA |
| *Hprt* Forward | TCATTATGCCGAGGATTTGGA |
| *Hprt* Reverse | GCACACAGAGGGCCACAAT |
| *Tbp* Forward | ACCTTATGCTCAGGGCTTGG |
| *Tbp* Reverse | GCCGTAAGGCATCATTGGAC |
